# Microglial depletion and activation: A [^11^C]PBR28 PET study in nonhuman primates

**DOI:** 10.1186/s13550-017-0305-0

**Published:** 2017-07-24

**Authors:** Ansel T. Hillmer, Daniel Holden, Krista Fowles, Nabeel Nabulsi, Brian L. West, Richard E. Carson, Kelly P. Cosgrove

**Affiliations:** 1 Department of Radiology and Biomedical Imaging, Yale University School of Medicine, New Haven, CT 06520 USA; 2 Department of Psychiatry, Yale University School of Medicine, New Haven, CT USA; 3 Yale PET Center, Yale University School of Medicine, New Haven, CT USA; 4 Plexxikon Inc, Berkeley, CA USA; 5 Department of Biomedical Engineering, Yale University School of Medicine, New Haven, CT USA; 6 Department of Neuroscience, Yale University School of Medicine, New Haven, CT USA

**Keywords:** Microglia, Inflammation, PET, Imaging, Immunology

## Abstract

**Background:**

The 18-kDa translocator protein (TSPO) is an important target for assessing neuroimmune function in brain with positron-emission tomography (PET) imaging. The goal of this work was to assess two [^11^C]PBR28 imaging paradigms for measuring dynamic microglia changes in *Macaca mulatta*.

**Methods:**

Dynamic [^11^C]PBR28 PET imaging data with arterial blood sampling were acquired to quantify TSPO levels as [^11^C]PBR28 *V*_T_. Scans were acquired at three timepoints: baseline, immediately post-drug, and prolonged post-drug.

**Results:**

In one animal*,* a colony-stimulating factor 1 receptor kinase inhibitor, previously shown to deplete brain microglia, reduced [^11^C]PBR28 *V*_T_ in brain by 46 ± 3% from baseline, which recovered after 12 days to 7 ± 5% from baseline. In a different animal*,* acute lipopolysaccharide administration, shown to activate brain microglia, increased [^11^C]PBR28 *V*_T_ in brain by 39 ± 9% from baseline, which recovered after 14 days to −11 ± 3% from baseline.

**Conclusions:**

These studies provide preliminary evidence of complementary paradigms to assess microglia dynamics via in vivo TSPO imaging.

## Background

The immune system in the central nervous system is critical to maintaining homeostasis in the brain. Imbalances in the brain’s immune system are linked to a variety of neurological pathologies [1]. Microglia are resident macrophages in the central nervous system that function as critical regulators of neuroimmune function. Microglia are quiescent under normal conditions [2]; however, microglia become “activated” in response to cytokines or other substances indicating the presence of neuroinflammation. Microglial activation is a necessary repair process, but dysregulated microglial function can cause neuronal dysfunction and cell death [3]. Thus, pharmaceutical therapeutics targeting microglia are of high interest to regulate neuroimmune response in many central nervous system disorders.

The colony-stimulating factor 1 receptor (CSF1R) is a tyrosine kinase of interest for regulating neuroimmune response. The endogenous ligands CSF1 and interleukin-34 (IL-34) act at the CSF1R, altering the activation and proliferation of microglia. Mice born without the *Csf1r* gene do not develop microglia and rarely survive to adulthood [4], while extensive treatment of rodents with CSF1R-selective inhibitors eliminated nearly all microglia in the brain [5]. Indeed, CSF1R inhibitors improved behavioral measures in a mouse model of Alzheimer’s disease [6] and reduced tumor-associated macrophage levels [7]. Moreover, microglia are the only brain cells expressing CSF1R [4], making drugs targeting CSF1R “microglial selective” in the brain. Assessment of microglia with molecular imaging could provide a critical tool evaluating important physiological effects of CSF1R treatments for psychiatry and neurodegenerative disorders.

Positron-emission tomography (PET) imaging of the 18-kDa translocator protein (TSPO) is a current state-of-the-art technique for the in vivo measurement of neuroinflammation. TSPO primarily is expressed on the outer mitochondrial membrane of microglia and reactive astrocytes, with low expression levels in other neuronal cells [8]. Additionally, TSPO levels are expressed at high levels when microglia are activated [9]. PET imaging with TSPO-specific radioligands such as [^11^C]PBR28 (*N*-acetyl-*N*-(2-[^11^C]methoxybenzyl)-2-phenoxy-5-pyridinamine) is commonly interpreted as a measure of activated microglia, although astrocytes and other neuronal cells expressing TSPO also contribute to the signal*.* Recent studies demonstrated that acute administration of *Escherichia coli* lipopolysaccharide (LPS), which triggers robust microglial activation, increased [^11^C]PBR28 volumes of distribution (*V*_T_) by 30–60% from baseline values in both baboons [9] and humans [10], which immunohistochemistry revealed to result almost exclusively in microglia [9]. However, the prolonged effects of acute LPS on TSPO imaging measures are unknown. Moreover, measurements of TSPO levels following microglial depletion have not been performed; such studies would provide a powerful complement to measurement of microglial response. The goal of this preliminary report is to assess two complementary [^11^C]PBR28 PET imaging paradigms for measuring dynamic changes in neuroinflammation, including microglial depletion with a CSF1R inhibitor and microglial activation via LPS administration, at both acute and prolonged time periods in *Macaca mulatta*.

## Methods

Two *Macaca mulatta* animals (both male, 6–7 years, 11–17 kg) were imaged.

PLX3397 [11], a selective CSF1R inhibitor (provided by Plexxikon Inc., Berkeley, CA), was made available in the animal’s daily biscuit allotment and in preferred foods at 165 mg/kg/day for seven consecutive days (15–21 days after baseline [^11^C]PBR28 scan). This dosing regimen robustly reduced brain microglia in rodents in a previous report^5^. However, not all the food was consumed. Therefore, on the following day (22 days after baseline scan), an additional oral gavage of 165 mg/kg PLX3397 was administered 2 h prior to the second [^11^C]PBR28 PET scan. A third [^11^C]PBR28 PET scan was acquired 12 days after the second [^11^C]PBR28 PET scan to assess recovery of TSPO levels.

LPS (NIH Clinical Center Reference Endotoxin *E. coli* serotype O:113) was used to induce microglial activation as shown previously [9, 10]. In a second animal, a baseline [^11^C]PBR28 scan was first acquired. After completion of the baseline scan, 1 ng/kg LPS (the dose given in the previous human imaging study [10]) was administered as a slow bolus over 1 min. A second [^11^C]PBR28 scan was acquired, starting 2.5 h after LPS administration to coincide with maximal neuroinflammatory and cytokine response [9, 10], in order to examine the acute effects of LPS on microglial activation. A third [^11^C]PBR28 PET scan was acquired 14 days after LPS administration to assess prolonged effects of LPS on microglial activation.

[^11^C]PBR28 was produced at high specific activity (> 120 MBq/nmol) as previously described [9]. Injected PBR28 masses were less than 1.54 nmol (range 0.16–1.54 nmol). At least 2 h before PET scanning, animals were initially anesthetized with ketamine hydrochloride (10 mg/kg, i.m.), and maintained on anesthesia with 1.5–2.5% isoflurane throughout the scan. Two catheters were placed, one in a saphenous vein for radiotracer administration and a second in a radial or femoral artery for blood sampling. Vital signs were continuously monitored for the duration of scanning procedures. PET imaging data were acquired with a Focus 220 (Siemens/CTI, Knoxville, TN). Data acquisition in list-mode began simultaneously with a slow 3-min bolus injection of 177–183 MBq [^11^C]PBR28, and continued for 120 min. To measure the metabolite-corrected [^11^C]PBR28 input function and free fraction (*f*_P_), arterial blood samples were manually acquired with the same sampling and analysis methods as previously described for nonhuman primates [9]. For region of interest identification, a T1-weighted MR image was acquired separately with a Siemens 3 T Trio scanner using an extremity coil and the following spin echo sequence: [echo time (TE) = 3.34 ms, repetition time (TR) = 2530 ms, flip angle = 7°, thickness = 0.50 mm, field of view = 140 mm]. Non-brain structures were removed using FMRIB’s Brain Extraction Tool.

List-mode PET data were histogrammed into time bins up to 5 min long and reconstructed with Fourier rebinning followed by 2-D filtered back projection with a 0.15-mm^−1^ Shepp filter. Reconstructed PET images were registered to each animal’s MR image, and the animal’s MR image was then normalized to a MRI atlas using the nonlinear registration algorithm in BioImage Suite 3.01. Time-activity curves were extracted from the caudate, cerebellum, cingulate cortex, frontal cortex, hippocampus, occipital cortex, putamen, temporal cortex, and thalamus. The primary imaging outcome measure was [^11^C]PBR28 total distribution volume (*V*_T_) [12], calculated using multilinear analysis method (MA1) [13] with t* = 15 min. Values of *V*_T_ were estimated for all regions of interest for primary analyses and further estimated at the voxel level to create parameterized maps for visual assessment.

## Results and discussion

**Fig. 1:**
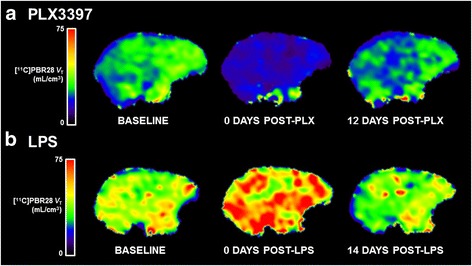
Imaging microglial depletion and activation with [^11^C]PBR28 PET. **a** Parametric images of [^11^C]PBR28 *V*_T_ at baseline (*left*), acutely post-PLX3397 (microglial depletion, *center*), and 12 days post-PLX3397 (recovery, *right*). **b** Parametric images of [^11^C]PBR28 *V*_T_ in a different animal at baseline (*left*), 2.5 h post-LPS (microglial activation, *center*), and 14 days post-LPS (recovery, *right*). All *V*_T_ parametric images are in units of milliliters per cubic centimeter

Administration of PLX3997 robustly reduced [^11^C]PBR28 *V*_T_. Immediately following 8 days of PLX3397 intake (7 days of food plus the oral gavage), which resulted in blood levels of 2.06 μg/mL PLX3397, [^11^C]PBR28 *V*_T_ was reduced by 46 ± 3% (averaged across regions) compared to baseline values (Table 1). Twelve days later, [^11^C]PBR28 *V*_T_ values were 7 ± 5% lower (averaged across regions) than baseline values (Fig. 1a).

**Table 1:** [^11^C]PBR28 experimental results

Study	Measurement	Caudate	Putamen	Hippocampus	Cingulate cortex	Frontal cortex	Occipital cortex	Temporal cortex	Cerebellum	Thalamus	*f*_P_ (%)
Baseline Pre-PLX	*V*_T_ (mL/cm^3^)	33.2	29.1	30.0	33.6	30.7	24.5	32.3	23.3	26.9	7.8
Immediate Post-PLX	*V*_T_ (mL/cm^3^)	16.6	15.1	17.3	16.9	16.1	13.7	18.3	12.8	14.6	5.7
	% Change from BL	−50%	−48%	−42%	−50%	−48%	−44%	−43%	−45%	−46%	−27%
12-day Post-PLX	*V*_T_ (mL/cm^3^)	26.4	26.8	28.4	31.7	29.3	23.4	30.0	22.8	24.8	7.9
	% Change from BL	−20%	−8%	−5%	−6%	−4%	−4%	−7%	−2%	−8%	1%
Baseline Pre-LPS	*V*_T_ (mL/cm^3^)	52.4	40.7	49.4	45.4	48.9	48.4	47.9	50.9	45.5	8.2
Baseline Post-LPS	*V*_T_ (mL/cm^3^)	71.4	58.4	74.3	67.1	64.3	59.0	66.9	68.5	65.7	11.3
	% Change from BL	36%	44%	50%	48%	32%	22%	40%	35%	44%	38%
2-week Post-LPS	*V*_T_ (mL/cm^3^)	45.4	35.7	45.5	39.0	42.9	41.5	44.4	47.3	39.8	8.0
	% Change from BL	−13%	−12%	−8%	−14%	−12%	−14%	−7%	−7%	−13%	−2%

In contrast, acute administration of 1 ng/mg LPS robustly increased [^11^C]PBR28 *V*_T_ by 39 ± 9% (averaged across regions) relative to baseline values (Table 1). Fourteen days later, [^11^C]PBR28 values were 11 ± 3% lower (averaged across regions) than baseline *V*_T_ values (Fig. 1b). Taken together, these data demonstrate the potential for two complementary imaging paradigms that assay dynamic changes in TSPO levels in vivo.

The reduction of [^11^C]PBR28 *V*_T_ following administration of PLX3397 is consistent with previous reports of microglial depletion throughout the brains of mice following administration of CSF1R inhibitors [5]. While TSPO is expressed in cells other than microglia such as astrocytes and endothelial cells, CSF1R inhibitors are specific to microglia in brain [4], and astrocyte morphology remained unchanged following CSF1R inhibition in rodents [5]. Therefore, the observed reduction in [^11^C]PBR28 *V*_T_ most likely results from depletion of microglia expressing TSPO. Previous TSPO saturation studies in rhesus monkeys demonstrated that over 95% of [^11^C]PBR28 *V*_T_ is attributed to specific binding [14], thus the 46 ± 3% reduction in [^11^C]PBR28 *V*_T_ could result from a partial depletion of microglia, a reflection of the astrocyte contribution to baseline *V*_T_ values, or a combination of the two*.* The small differences (7 ± 5%) in [^11^C]PBR28 *V*_T_ between baseline and 12 days after the course of PLX3397 are consistent with reported recovery of microglial levels after termination of CSF1R inhibition [5]. Our findings here suggest that TSPO imaging with PET could be used to measure microglial depletion via drugs such as CSF1R inhibitors in vivo.

The increase of [^11^C]PBR28 *V*_T_ following the administration of 1 ng/kg LPS is also consistent with previously reported increase (30–60%) of [^11^C]PBR28 *V*_T_ 3 h following administration of the same LPS dose in humans [10]. Similar results were also reported for baboons, where 29% and 62% increases in [^11^C]PBR28 *V*_T_ were reported following 1 and 4 h, respectively, post administration of 0.1 mg/kg LPS [9]. The previous baboon study acquired a further [^11^C]PBR28 scan 22 h after LPS administration in two animals to examine sub-acute LPS effects. In those scans, [^11^C]PBR28 *V*_T_ was reduced by 43% compared to baseline in one animal, whereas [^11^C]PBR28 *V*_T_ returned to baseline in the second animal. It is unclear whether prolonged anesthesia may have influenced those results. The present data further suggest that TSPO levels recover by 14 days following LPS-induced activation, as [^11^C]PBR28 *V*_T_ values were 11% lower than baseline at this time. While additional studies are needed for confirmation, these data support the hypothesis that TSPO levels recover over an extended time after LPS administration. While the test-retest reproducibility of [^11^C]PBR28 *V*_T_ in rhesus monkey has not formally been reported, the test-retest variability in humans at our imaging center is 8% in gray matter regions [15]. Thus the differences in our measured [^11^C]PBR28 *V*_T_ values at extended imaging timepoints relative to baseline are likely consistent with test-retest variability.

Previous studies noted that LPS administration reduced [^11^C]PBR28 concentrations in arterial plasma. Here, a similar effect was observed following LPS administration. A possible explanation for this behavior is increased specific uptake of radiotracer not only in brain but throughout the periphery, reducing parent radiotracer concentrations available in arterial plasma. Following PLX3397 administration, [^11^C]PBR28 concentrations in arterial plasma increased from baseline by roughly 50%, while tissue concentration in the brain also decreased from baseline by roughly 25%, resulting in a net decrease of 46% in *V*_T_. Similarly, this net increase in [^11^C]PBR28 concentrations in arterial plasma could have resulted from less specific uptake of radiotracer throughout the periphery following chronic PLX3397 administration. In addition, *f*_P_ changed in the acute scan from both paradigms. Following 1 ng/kg LPS administration, *f*_p_ increased by 38%, contrasting previous human studies where *f*_P_ remained unchanged following the same LPS dose [10]. Following CSF1R inhibition with PLX3397, *f*_P_ decreased from 7.8 to 5.7%; however, *V*_T_/*f*_P_ values were still 27 ± 4% lower than baseline. Importantly, the PLX3397 dosing schedule did not affect blood-brain barrier (BBB) integrity in rodents [5], thus is it unlikely that the reported *V*_T_ values were biased by a compromised BBB. To summarize, because administration of PLX3397 or LPS results in complex effects on [^11^C]PBR28 behavior in both the blood and the tissue, we contend that *V*_T_ should be used as the outcome measure to fully capture the changes in brain levels of TSPO with this imaging paradigm.

This report is not without caveats. Only one animal was imaged for each of the microglial activation and microglial depletion paradigms. A larger sample size would improve confidence in these findings, yet we believe that the robust findings and within subject design provide preliminary proof of principle for this imaging paradigm. In addition, the interpretation of microglial depletion following PLX3397 administration is inferred from prior rodent studies instead of direct determination using methods such as immunohistochemistry or post mortem protein binding. Finally, the exact dose of PLX3397 received by the animal is not known, as not all the food was consumed. Therefore the timing and oral dose of CSF1R inhibitor for 46 ± 3% reduction of [^11^C]PBR28 *V*_T_ in rhesus monkey is unclear.

## Conclusions

We present data preliminarily indicating two complementary TSPO imaging paradigms allowing for dynamic in vivo measurements of microglial depletion and microglial activation. These techniques are of great interest for studying neuroinflammation in neurodegenerative and psychiatric disorders, and for the evaluation of therapeutics targeting microglia in the brain.
